# The use of the roter interaction analysis system in assessing veterinary student clinical communication skills during equine wellness examinations in rural Kentucky, USA: A pilot study

**DOI:** 10.1002/vro2.23

**Published:** 2021-11-24

**Authors:** Mary Mauldin Pereira, Elpida Artemiou, Pedro De Pedro, Cindy Adams, Caroline Ritter

**Affiliations:** ^1^ Department of Clinical Sciences Ross University School of Veterinary Medicine Basseterre St. Kitts; ^2^ Department of Biomedical Sciences Ross University School of Veterinary Medicine Basseterre St. Kitts; ^3^ Department of Veterinary Clinical and Diagnostic Services Faculty of Veterinary Medicine University of Calgary Calgary Canada; ^4^ Department of Health Management Atlantic Veterinary College University of Prince Edward Island Charlottetown Canada

**Keywords:** communication research, equine health, RIAS, veterinary communication, veterinary education

## Abstract

**Background:**

Effective clinical communication can aid veterinarians in building good client relationships, increase adherence to recommendations and, ultimately, improve patient health and welfare. However, available information on veterinary communication in the equine context is limited. The objective of this study was to describe the communication of veterinary students in the equine environment who had previous communication training. Additionally, we assessed the suitability of the Roter Interaction Analysis System (RIAS) for the analysis of audio‐video recordings of equine wellness consultations.

**Methods:**

Twenty‐seven equine wellness consultations performed by second‐year Ross University School of Veterinary Medicine students were recorded in rural Kentucky, United States of America. Recordings were submitted to a professional coder who applied the RIAS to the equine context by expanding or adjusting code definitions.

**Results:**

A substantial amount of utterances (i.e. segments of speech) were allocated to core communication skills including building rapport (30%), facilitation and client activation (24%) and education and counselling (23%). There was a large variation in utterances used among consultations of the same veterinary student and students; they did not appear anxious or nervous.

**Conclusions:**

Students made use of core communication skills, indicating that experiences from pre‐clinical training could be transferred to equine practice. Furthermore, this study demonstrated that the RIAS could be considered for consecutive studies aiming to provide observational data on clinical communication in the equine context.

## INTRODUCTION

In human medicine, there is a substantial amount of literature that supports the importance of teaching and learning communication skills.^1^ Although research in the veterinary context has a growing literature base, effective clinical communication is a relatively recent topic in veterinary medicine aiming to improve client adherence and enhance outcomes for the patient.^2^, ^3^, ^4^ As such, the significance of clinical communication within veterinary medicine is developing a focus that addresses differences and similarities across companion as well as production animals.^3^, ^5^, ^6^ Animal owners are the caretakers in veterinary medicine,^7^ therefore, in both veterinary and human medicine, successful consultations are reliant on helpful and trusting interactions with humans.^3^, ^7^ In that regard, evidence within veterinary medicine literature identifies communication skills as a core clinical competency and validates its importance in achieving more effective and fulfilling consultations.^8^ Assessing patients’ quality of life requires clinical decision‐making as well as the usage of specific communication skills so that practitioners may acquire an accurate assessment of the owners’ perspective.^9^ Additionally, when effective clinical communication skills are utilized, the results of patient and client care are optimized.^8^ Examples of communication skills that positively contribute to the veterinarian‐client relationship, enhancing client and practitioner satisfaction, include involving the client in the consultation through open‐ended questions and building rapport (e.g. through empathic and partnership statements).^2^, ^10^, ^11^

It has been recommended that veterinary curricula include communication training such that veterinary graduates begin their careers with appropriate levels of competency in clinical communication.^12^, ^13^ Presently, the American Veterinary Medical Association Council on Education requires that all accredited colleges of veterinary medicine teach communication skills^13^; research in veterinary medicine has demonstrated that training can improve communication skills.^11^ However, the effectiveness of the training on veterinary students’ communication skills depends on a variety of demographic factors^14^ and the method of training delivery.^15^

Specifically, communication skills are being incorporated into the curricula of veterinary schools and taught using a variety of teaching methods which may include lectures, small group interactive sessions, role‐play and web‐based programmes.^8^, ^16^, ^17^ Lecturing on communication in classroom settings has restricted value whereas one‐on‐one or small group teaching and learning is fundamental and allows for sufficient opportunity to practice communication skills in a supportive environment.^18^ A framework frequently used in veterinary medicine is the Calgary Cambridge Guide (CCG), which is a consultation model adapted for veterinary education.^6^, ^11^, ^16^ The CCG is generally used as a practical teaching framework for educating students on communication skills^11^, ^16^ and the Roter Interaction Analysis System (RIAS) methodology can be used to assess the communication skills taught with the CCG framework.^2^, ^19^ The CCG outlines 73 communication skills that are organized through five consecutive stages and outlines that practitioners should provide structure and build a relationship with the client throughout the consultation.^11^, ^16^, ^20^

Veterinary communication skills have been explored in companion and production animal contexts, with a limited study conducted in equine settings.^8^, ^21^ Equines can be considered companion animals, although they can also be used for breeding, sport and recreation, competition as well as meat production. There is a varied range in the nature and intensity of interactions with equines, indicating that relationships between humans and equines are likely to differ depending on the specific context.^22^, ^23^ Communication skills do not necessarily change in different contexts and specific skill requirements surrounding the client's perspective, rapport, structure, gathering information, as well as explaining and planning remain unchanged regardless of the setting.^6^, ^11^ Though based on the nature of the visit, certain skills may be intensified or relied on more than others.^24^ Results from surveys and interviews demonstrated that there are gaps surrounding relationship‐centred care.^25^ The term relationship‐centred care is characterized by a mutual understanding of the pet's well‐being and encompasses respect for the client's perspective and concerns as well as their own knowledge for their pets’ care.^5^ This includes the importance of shared decision making and client expectations in equine veterinary medicine, which can potentially negatively affect client adherence and the veterinarian‐client relationship.^25^ Research evidence, purposely gathered under field conditions, regarding equine client expectations, veterinary communication skills and the veterinarian‐client relationship evidence is needed.^10^

A commonly used approach in analysing clinical consultations is through audio‐video recordings using the RIAS, which was initially developed for human medicine contexts.^26^, ^27^ Extending the use of the RIAS to the veterinary environment provides an added opportunity to compare clinical communication across animal contexts. The RIAS is considered a predictive and valid tool, which has been used successfully in veterinary communication studies, specifically in companion animal practice^2^, ^24^, ^28^, ^29^ and in dairy cattle practice.^21^ The RIAS is a measure of medical conversations where dialogue is broken down into utterances (i.e. the smallest segments of speech; words or phrases that constitute a complete thought or idea). This allows for the communication of both the practitioner and client/patient to be coded and quantified.^2^ The RIAS analysis can identify communication skills demonstrated as well as missed during the practitioner‐client interaction.^26^

The aims of this explorative study were to investigate and describe the communication skills of pre‐clinical veterinary students during equine wellness consultations who have undergone communication training delivered by Ross University School of Veterinary Medicine (RUSVM). Furthermore, to the best of the authors’ knowledge, this is the first study to explore the applicability of the RIAS in the equine context using audio‐video recordings captured under a variety of challenging environmental conditions in equine rural stables. Successful application of the RIAS in equine medicine would add communication information from another context despite contextual challenges, hence further establishing the RIAS as a beneficial tool for consistently measuring medical conversations.

## MATERIALS AND METHODS

### Participants

RUSVM students complete seven semesters of the pre‐clinical curriculum with three 15‐week semesters per year on the island of St. Kitts. The pre‐clinical curriculum communication skills training includes interactive lectures, web‐based learning as well as participation in small group interactions involving experiential practice with trained simulated clients portraying real‐life case scenarios representing most species. Mandatory clinical communication laboratories are held in semesters 3, 5, 6 and 7 with a total of nine hours of hands‐on communication practice received over their pre‐clinical semesters. The CCG is the framework used for teaching, learning and assessing students at RUSVM.

For this study, members of the Student Chapter of the American Association of Equine Practitioners (SCAAEP) were recruited. A RUSVM equine teacher held a meeting for second‐year pre‐clinical SCAAEP members. During the meeting, the RUSVM equine clinician provided participation details such as dates, locations, expectations and goals for the study. The students were given the opportunity to enrol in an equine practicum in which they could practice their clinical skills including clinical communication through hands‐on experiences. As part of this practicum, participants were informed that communication skills were being recorded and assessed. The equine clientele was recruited through informative meetings held by 4H (a youth development organization in the USA composed of more than 100 public universities which provides experiences for young people to learn by doing). Following this, the equine clientele were offered subsidized wellness examinations. Prior to participation, each student and client reviewed and signed an informed consent form. The study was approved by the RUSVM Institutional Review Board committee.

### Participant information

The second‐year pre‐clinical students, all females and of Caucasian ethnicity enrolled in their 6th semester, volunteered to participate to gain additional skills in performing equine wellness examinations as well as acquire communication experience under the supervision of the RUSVM equine clinician. All participating students successfully completed three communication laboratories and all simulated practice only included canine cases. The canine cases are included in the veterinary curriculum delivered to all enrolled RUSVM students. Each laboratory included one interaction with one simulated client totalling six hours of communication training. Clientele in this study were all English‐speaking owners of pleasure horses and Amish owners of working horses.

### Wellness equine examinations

The wellness equine examinations were scheduled during a RUSVM semester break (April 22nd–25th, 2017) and were organized over three consecutive days across Boyd, Hardin and Whitley counties at private barn facilities and fairgrounds in rural Kentucky. The RUSVM equine clinician worked in conjunction with the University of Kentucky equine clinicians to organize and offer these specific locations. One student led each of their individual interactions where the student and client consultation included (1) greeting, (2) gathering historical information, (3) performing a physical examination and (4) educating the client. For each student, there were between five to seven total interactions recorded. Of these, two students that had a total of seven recordings interacted with the same client twice. Although some consultations included the same client, each case consultation was different based on the patient. Case scenarios were similar across all patients as the consultations were centred around wellness examinations. Some of these consultations also included administering routine vaccinations, collecting blood for Coggins testing, assisting with dental examinations and collecting faecal material for required diagnostic tests.

### Audio‐visual recording

A hired audio‐visual team supported camera set up across all locations and outfitted students and clients with individual microphones for media capture (Images 1 and 2). The hired team included two audio technicians for camera operations per day, mileage for automobile travel to rural settings and four wireless microphone sets.

### Analysis of recordings

An experienced RIAS coder from Johns Hopkins Bloomberg School of Public Health (Baltimore, MD, USA) was hired and analysed all equine interactions following coding conventions established in earlier veterinary RIAS studies.^28^, ^30^ Specifically, the RIAS does not require transcription of recordings. Instead, mutually exhaustive and exclusive codes are directly assigned to each utterance spoken by the veterinary student or client, resulting in a quantitative dataset.^19^ Intra‐coder reliability was not required because the coder assigned to the study had demonstrated high levels of reliability in previous studies.^31^ Furthermore, the data set was small and re‐coding 10% of the interactions as done in a previous study^32^ would not have been informative.

For this study, we calculated summary measures (i.e. composites) from the codes assigned to the veterinary student talk (Table 1). Specifically, closed‐ended questions were assessed, in which the student asked questions that only required a short answer from the client (e.g. yes/no). Open‐ended questions were identified using a broader definition versus simply the grammatical format, which signalled the coder of the student's intent to probe and elicit additional information. Education and counselling statements included utterances that provided information or advice to the client. All questions as well as education and counselling utterances could be either of biomedical nature (e.g. related to sickness or treatment of the horse) or had to do with the horse's lifestyle (e.g. related to exercise or diet of the horse). Furthermore, facilitation and client activation statements were calculated, which, for example, included the veterinary student asking for the clients’ permission, opinion and understanding. The rapport building summary measure included, among others, utterances coded as reassurance, concern, empathy, self‐disclosure and agreement. Procedural talk comprised utterances related to transition statements or providing instructions.

**TABLE 1:** Examples of codes assigned to veterinary student utterances (i.e. smallest segments of speech) during equine wellness consultations using the Roter Interaction Analysis System (RIAS)

RIAS code	Examples of utterance
**Data gathering**	
**Open‐ended questions**	
Biomedical	On any medicine for that?
Lifestyle	Any differences in behaviour?
**Closed‐ended questions**	
Biomedical	How many times a day do you give it?
Lifestyle	Is she at grass?
**Education and counselling**	
Biomedical	You can give it to her twice a day.
Lifestyle	You can keep her active.
**Relationship building**	
Facilitation and client activation	Do you think we can hold her?
Rapport building	
Positive talk	I like him very much.
Emotional talk	I just worry.
Social talk	I've gotta check that out, there's a lot going on around here.
Negative talk	They weren't very nice.
**Procedural talk**	I am going to listen to her heart, can you grab this?

Additionally, the RIAS coder assigned global affect ratings to the student‐client interaction after each consultation representing their subjective impression of the overall character of the visit on six‐point semantic differential items (e.g. how rushed, friendly and nervous they perceived the veterinary student to be).^24^, ^32^, ^33^ Stata IC 15.0 (StataCorp. 2015. Version 14) was used to calculate summary measures based on the codes obtained from the RIAS coder.

## RESULTS

The RIAS coder applied communication codes to a total of 27 recorded interactions across five students. The length of the recorded consultations ranged from four to 17 min, with a mean and median recorded consultation length of 10 min. The audio quality of the 27 analysed consultations was rated by the RIAS coder as “good” with the exception of two recordings where the audio quality was rated as “fair”. In the same regard, only two of all veterinary utterances included in the consultations could not be coded because they were inaudible and not acoustically understandable by the RIAS coder (i.e. uniterable utterances). Three (11%) of the recordings had an abrupt beginning as the recording started while the interaction was already in progress, while 10 (37%) recordings had an abrupt end and one (4%) recording had both.

**FIGURE 1:**
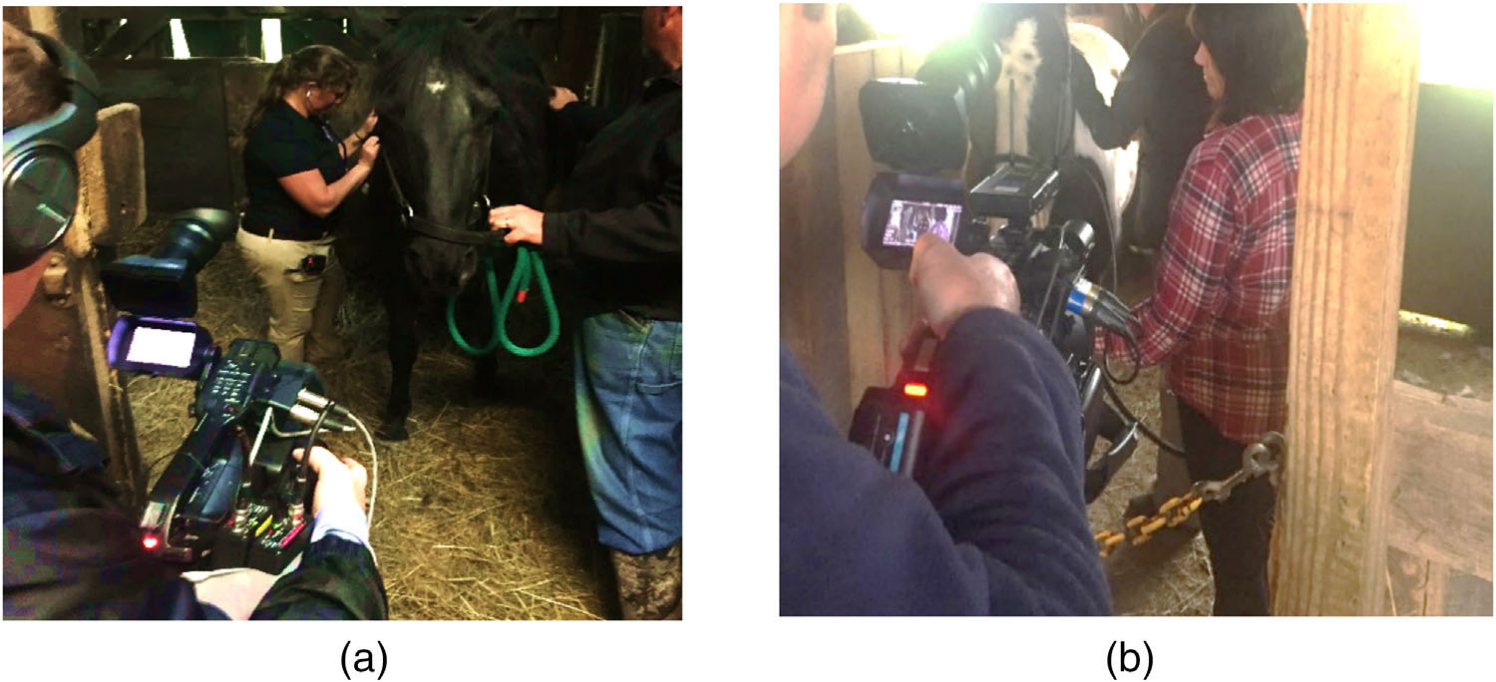
Student participants, clients, and hired audio‐visual team during equine wellness consultations

The RIAS analysis demonstrated that 30% of students allocated substantial amounts of their talk to building rapport (30% calculated as a mean across all recorded consultations (Figure 1) and 24% of students assigned their talk to facilitation and client activation). Furthermore, across all consultations, students dedicated 23% of their interaction to education and counselling, 9% to procedural talk, 8% to closed‐ended questions and 5% to open‐ended questions. Figure 1 also demonstrates that the percentage of talk allocated to specific communication skills varied among students. For example, Student 2 only allocated approximately 7% of all talk to ask questions, whereas Student 5 allocated approximately 20% to this task.

A substantial variation in the absolute number of utterances dedicated to a specific communication composite among recordings of the same student was noted, even when recordings were similar in length (Table 2). For example, recorded consultations ranged from 9–11 min for Student 4, however, this student used only six statements educating and counselling the client in one consultation but 41 statements in a different consultation. Despite the large variation among consultations of the same veterinary student and students, it appeared that they generally made use of open‐ended questions, involving the client through facilitation and activation, as well as building rapport. In contrast, the use of negative talk was very rare (Figure 1, Table 2). Despite the large differences in the number of utterances used by the students, the variation in the global affect ratings was minimal (Table 3). Students did not appear angry, irritated, anxious or nervous in any of the consultations. For the other categories, all ratings ranged between 3 (slightly below the baseline rating) and 4 (slightly above the baseline rating) which resulted in low standard deviations.

**TABLE 2:** Veterinary student communication composites during 27 equine consultations analysed using the Roter Interaction Analysis System

	**Communication utterances: Mean (min–max)**					
**Communication Composite**	**Student 1**	**Student 2**	**Student 3**	**Student 4**	**Student 5**	**All Students**
**Data gathering**	**14 (2–22)**	**8 (2–21)**	**14 (6–25)**	**11 (8–13)**	**18 (10–34)**	**13 (2–34)**
Open‐ended questions	5 (1–11)	3 (1–10)	3 (0–6)	3 (0–4)	9 (6–18)	5 (0–18)
Closed‐ended questions	9 (1–17)	5 (0–11)	11 (6–21)	8 (5–11)	10 (3–16)	8 (0–21)
**Education and counselling**	**41 (12–87)**	**34 (4–87)**	**14 (4–22)**	**22 (6–41)**	**19 (4–52)**	**28 (4–87)**
**Relationship building**	**74 (28–119)**	**65 (21–121)**	**47 (21–66)**	**55 (45–74)**	**56 (23–97)**	**61 (21–121)**
Facilitation and client activation	28 (11–50)	34 (6–70)	21 (7–38)	26 (7–46)	27 (10–59)	28 (6–70)
Rapport building	46 (17–73)	30 (15–51)	26 (14–42)	29 (13–45)	30 (13–45)	33 (13–73)
Positive talk	26 (10–40)	14 (6–24)	15 (10–21)	15 (7–22)	17 (9–32)	18 (6–40)
Emotional talk	17 (3–37)	12 (2–22)	6 (4–9)	9 (6–15)	10 (2–25)	11 (2–37)
Social talk	3 (0–4)	5 (0–10)	4 (0–15)	5 (0–18)	2 (0–4)	4 (0–18)
Negative talk	0.1 (0–1)	0 (0–0)	0 (0–0)	0.5 (0–1)	0 (0–0)	0.1 (0–1)
**Procedural talk**	**15 (2–26)**	**7 (2–15)**	**12 (7–23)**	**7 (1–14)**	**10 (3–25)**	**10 (1–26)**
Uniterable utterances^a^	0 (0–0)	0 (0–0)	0 (0–0)	0.5 (0–2)	0 (0–0)	0.1 (0–2)
**Utterances total**	**144 (51–199)**	**114 (35–209)**	**87 (60–111)**	**95 (77–110)**	**104 (40–187)**	**112 (35–209)**
Length of recorded consultation (min)	10 (4–16)	10 (4–17)	9 (7–13)	10 (9–11)	9 (5–15)	10 (4–17)
Analysed consultations (#)	7	6	5	4	5	27

^a^ Utterances (i.e. the smallest segment of speech) that the Roter Interaction Analysis System coder could not understand acoustically.

Mean use of utterances was calculated across several (i.e. 4–7) consultations of the same student.

**TABLE 3:** Global affect ratings of veterinary students’ equine consultations

	**Global affect ratings (median [min–max])** **^a^**					
	**Student 1**	**Student 2**	**Student 3**	**Student 4**	**Student 5**	**Total**
Anger/Irritation^b^	1 (1–1)	1 (1–1)	1 (1–1)	1 (1–1)	1 (1–1)	1 (1–1)
Anxiety/Nervousness^b^	1 (1–1)	1 (1–1)	1 (1–1)	1 (1–1)	1 (1–1)	1 (1–1)
Dominance/Assertiveness^c^	4 (4–4)	4 (4–4)	4 (4–4)	3 (3–3)	4 (4–4)	4 (3–4)
Interest/Attentiveness^c^	4 (4–4)	4 (4–4)	4 (4–4)	4 (4–4)	4 (4–4)	4 (4–4)
Friendliness/Warmth^c^	4 (4–4)	4 (4–4)	4 (4–4)	4 (4–4)	4 (4–4)	4 (4–4)
Responsiveness/Engagement^c^	4 (3–4)	4 (4–4)	4 (3–4)	3 (3–3)	4 (4–4)	4 (3–4)
Sympathetic/Empathetic^c^	4 (4–5)	4 (4–5)	4 (4–4)	3.5 (3–4)	4 (4–4)	4 (3–5)
Hurried/Rushed^c^	3 (3–3)	3 (3–3)	3 (3–3)	3 (3–3)	3 (3–3)	3 (3–3)
Respectfulness^c^	4 (4–4)	4 (4–4)	4 (4–4)	4 (4–4)	4 (4–4)	4 (4–4)
Interactivity^c^	4 (4–4)	4 (4–4)	4 (4–4)	4 (4–4)	4 (4–4)	4 (4–4)

Several (i.e. 4–7) consultations per student were rated by the Roter Interaction Analysis System coder.

^a^ Measured on 6‐point semantic differential items with 1 being the lowest and 6 being the highest rating.

^b^ Baseline was a rating of 1 with a rating >1 indicating increased anger/irritation or anxiety/nervousness.

^c^ Baseline was a rating of 3.5 with a rating <3.5 and >3.5 indicating decreased and increased veterinary display of the particular measure, respectively.

## DISCUSSION

To the best of the authors’ knowledge, this is the first study to explore the application of RIAS to an equine setting. This study demonstrated the successful capture of audio‐video recordings and RIAS in the equine context to analyse veterinary student communication skills that were demonstrated with clientele, as compared to simulated clients. The audio‐visual recordings were of high quality despite the exposure to often challenging environments. Examples of challenges posed included rainy conditions and limited stable spaces to accommodate interactions for the students, clients and veterinarians. Furthermore, the RIAS coder was successful in applying the communication codes that were initially developed for the human medicine context to the equine environment by expanding or adjusting the code definitions. For example, similar to the human context, codes related to the “lifestyle” of the patient (i.e. horse) included diet or exercise routine but, additionally, whether or not the horse has access to pasture. This approach provided a comprehensive dataset that can be used as a baseline to design future similar studies. Similar to a previous study,^24^ future research could describe communication skills during equine consultations when the students or practitioners discuss specific health problems rather than conduct wellness examinations. Furthermore, investigation of the association of specific student attributes to communication skills will provide additional information on underlying factors for successful consultations.

The audio quality of the 27 analysed consultations was rated by the RIAS coder as “good” with only two recordings being rated as “fair” despite the consultations taking place in different locations during adverse weather conditions, background noise and physical distances between the camera and student‐client interaction. This indicates that the technical audio‐video recording was suitable for providing high‐quality recordings with a lower number of uniterable utterances compared to the use of action cameras worn by dairy practitioners in a previous study.^21^ Based on the results, it could be posed that there was likely a minimal influence of accent on the RIAS analysis, however, investigators in future studies could consider this in their study design. However, depending on the context and setting of the study, hiring an audio‐video team might not always be practical or feasible. Besides the additional expenses of hiring an audio‐visual team, multiple abruptions of recordings were observed. Identifying an automated system to ensure the correct beginning and ending times could result in less abruptions.

The data derived from the 27 analysed recordings should be interpreted with caution due to the relatively small sample size and as such generalizability can be limited. Another limitation is the high number of recordings with abrupt beginnings or ends. The abrupt beginning and endings may have impacted the captured skills, specifically those that are offered with greeting and closing the consultation. However, all students used a considerable number of statements related to rapport building, facilitation and client activation, together contributing to over half of the total talk.

Although a previous report^29^ used slightly different summary measures when assessing veterinary communication in the companion animal context, the percentage of talks dedicated to rapport building in this study was similar and was also comparable to interactions of dairy veterinarians and farmers.^32^ In contrast, 24% of all equine veterinary student talk was coded as facilitation and client activation as compared to <10% reported in the companion and dairy farm context.^29^, ^32^ Use of such statements is intentionally taught and practised in the RUSVM communication curriculum. Therefore, a possible explanation for the relatively high use of these communication skills may have to do with the explicit communication skills training that these students received during their pre‐clinical experiential sessions. However, if the communication training indeed increased the use of specific communication skills, this should be determined using randomised controlled trials, as previously reported in human medicine^34^ and suggested in companion animal settings.^2^, ^15^ Furthermore, larger sample size is required to draw more generalizable conclusions.

The pre‐clinical communication training supported students in successfully demonstrating several core communication skills. Specific skills supporting relationship‐building were intentionally practised during the interactions and previous studies indicate that such can improve the possibility that the client will share their perspective as well as follow recommendations provided.^10^, ^18^ The amount of relationship‐building talk was fairly consistent across all students, whereas other utterances varied greatly. Especially, the number of questions asked differed between students and consultations. It has to be acknowledged that the use of specific communication skills (e.g. open‐ended questions) will depend on the presented equine case, hence, similarly to previous findings,^21^ it is advised to record several consultations per practitioner to obtain a more accurate estimate of the communication skills. However, in this study, even averages of communication skills used across several recordings varied depending on the student. Therefore, this study provides evidence that there is variation across different students as well as within the same student. Repeated interactions involving the same client may have impacted specific aspects of communication, however, these interactions were few and independent of the client based on the presented patient.^8^

There was substantial observed variability in utterances, despite having a relatively small dataset. In contrast, variation in the global affect ratings was minimal. Therefore, while global affect ratings were suitable to describe the overall impressions the RIAS coder had of how students interacted with clients, it is perhaps not sensitive enough to identify subtle nuances in the conversation dynamics and provided limited opportunity to contrast different interactions.

Students did not appear angry, irritated, anxious or nervous. This lack of anxiousness and nervousness was unforeseen because they were being video‐recorded and observed by a professional team. Several factors may have led to these findings. First, the wellness examinations do not pose a high risk for the patient or the client. Second, procedural costs for equine wellness examinations for this study were standardised, minimizing discussions surrounding financial concerns. Third, the environment was well controlled and organized; providing a safe environment for students, clients and horses. Last, each SCAAEP student participant had previous experience working with both equine clientele and patients.

In addition to providing a comprehensive quantitative dataset suitable for detailed analyses,^26^ the RIAS can be a useful tool to compare communication skills to the elements of the CCG because of similar categorisation (e.g. relationship building is a component in the CCG and the RIAS). However, it has to be acknowledged that there are other means of evaluating clinical communication skills. Especially, as the assessment of motivational interviewing skills using the Motivational Interviewing Treatment Integrity Code (MITI) has gained traction in veterinary medicine.^35^, ^36^ Motivational interviewing is a process‐oriented technique aiming at facilitating clients' internal motivation to change by using specific communication skills to help clients resolve ambivalence.^37^ Both methods have their specific applications and researchers have to decide which approach suits their purposes when designing studies.

The RIAS successfully identified, categorized and coded utterances spoken by pre‐clinical veterinary students and equine clients. Students used core communication skills taught as part of their pre‐clinical curriculum. Audio‐video recordings and the RIAS are appropriate and applicable tools to capture and analyse clinical communication skills presented in equine rural settings. Hiring a professional audio‐visual team allowed for an overall good capture quality despite the variety of locations, weather conditions, and physical distances of clients and students. Larger sample size is needed for future studies to obtain more well‐defined conclusions surrounding clinical communication skills within an equine context.

## CONFLICT OF INTEREST

The authors declare that they have no conflicts of interest.

## ETHICS STATEMENT

The study met all ethical requirements and was approved by the Ross University School of Veterinary Medicine (RUSVM) Institutional Review Board (IRB).

## Data Availability

The study data including raw data, processed data, software, methods and materials can be made available upon request.
